# Adaptive Attention Memory Graph Convolutional Networks for Skeleton-Based Action Recognition

**DOI:** 10.3390/s21206761

**Published:** 2021-10-12

**Authors:** Di Liu, Hui Xu, Jianzhong Wang, Yinghua Lu, Jun Kong, Miao Qi

**Affiliations:** 1 College of Information Sciences and Technology, Northeast Normal University, Changchun 130117, China; liud737@nenu.edu.cn (D.L.); xuh504@nenu.edu.cn (H.X.); wangjz019@nenu.edu.cn (J.W.); luyh@nenu.edu.cn (Y.L.); 2 Institute for Intelligent Elderly Care, Changchun Humanities and Sciences College, Changchun 130117, China; 3 Key Laboratory for Applied Statistics of MOE, Northeast Normal University, Changchun 130024, China

**Keywords:** graph convolutional networks, action recognition, attention, skeleton sequence

## Abstract

Graph Convolutional Networks (GCNs) have attracted a lot of attention and shown remarkable performance for action recognition in recent years. For improving the recognition accuracy, how to build graph structure adaptively, select key frames and extract discriminative features are the key problems of this kind of method. In this work, we propose a novel Adaptive Attention Memory Graph Convolutional Networks (AAM-GCN) for human action recognition using skeleton data. We adopt GCN to adaptively model the spatial configuration of skeletons and employ Gated Recurrent Unit (GRU) to construct an attention-enhanced memory for capturing the temporal feature. With the memory module, our model can not only remember what happened in the past but also employ the information in the future using multi-bidirectional GRU layers. Furthermore, in order to extract discriminative temporal features, the attention mechanism is also employed to select key frames from the skeleton sequence. Extensive experiments on Kinetics, NTU RGB+D and HDM05 datasets show that the proposed network achieves better performance than some state-of-the-art methods.

## 1. Introduction

Research on human action recognition has become one of the most active issues in the computer vision area in recent years. It has been widely used in security surveillance [1], robot vision [2], motion-sensing games, virtual reality [3], etc. The existing methods [4,5,6] for action recognition are often conducted on RGB video or skeleton data. Although these methods have impressive performance, they are often affected by dynamic circumstances, viewpoints, illumination changes and complicated backgrounds, etc.

A human skeleton sequence can express discriminative and robust dynamic information for action recognition tasks. Skeleton data is composed of coordinates of human body key joints. It can effectively describe the body structure and dynamic of actions. Due to the absence of color information, the skeleton data is more robust to the variants of appearance and environment and more efficient in computation [7,8]. In addition, skeleton data is extremely compact in terms of data size. It is not only an effective representation of human body structure but also easy to be obtained by pose estimation algorithms [9,10] or depth sensors, such as Microsoft Kinect, Asus Xtion and Intel RealSense. Because of the development of these low-cost sensors, they are widely used in many fields to capture the motion of human joints. These sensors could also acquire different modalities of data, such as depth and RGB video.

Earlier skeleton-based methods [11,12,13,14,15,16] usually arranged human body joints as a chain and employed their coordinates as a vector sequence. Considering the sequence property, Recurrent Neural Networks (RNNs) are naturally applied to the action recognition task [11,12,15]. On the other hand, some works considered the skeleton data as a pseudo-image. Then Convolutional Neural Networks (CNNs) were employed to recognize the actions [13]. However, these methods ignore the latent spatial structure of the body, which is beneficial for action recognition. Due to the topological structure of the human skeleton, modeling skeleton data as a graph is more suitable than a vector sequence or pseudo-image [17,18,19,20]. Conventional deep learning methods [21,22,23] that conduct convolution on graphs have shown impressive success in many applications. Inspired by the truth that human skeleton data is naturally a topological graph, Yan et al. [20] first introduced Graph Convolutional Networks (GCNs) [21] and proposed a spatial-temporal graph convolutional network (ST-GCN) for the action recognition task based on the human skeleton. They constructed a spatial-temporal graph and conducted convolution on it. After that, many GCN-based methods [17,18,19,24,25,26] were proposed and achieved superior performance than traditional methods. Nevertheless, the graph structures in most of the GCN-based methods are constructed manually and would be fixed during the training process. For instance, the skeleton graph in [20] merely represented the physical structure of the human body. The relations of joints would be built only in a local context. It may not be suitable for some actions that happened between the joints that are not directly connected with physical connection, such as “clapping”, “talking on the phone”, “comb one’s hair”, etc. To capture the relations between distant joints, some approaches extracted multi-scale structural features via higher-order polynomials of the skeleton adjacency matrix. Li et al. [17] introduced multiple-hop modules to establish correlations between distant joints. However, it still had limitations for establishing long-range joints dependencies. On the other hand, there were redundant connections between further and closer neighbors. To capture both nearby joints dependencies and distant joints relations, we designed a simple yet efficient adaptive adjacent matrix.

In addition, some works [19,20,27] simply conducted 2D convolution on temporal dimensions that cannot effectively capture long-range temporal features. Examples such as “put on hat” and “take off hat” are very ambiguous in a short time observation. It requires capturing long-range temporal information to distinguish them. Therefore, we construct a memory module to remember the long-range temporal information by GRU. Furthermore, not only what happened in the past is critical, but also the information in the future is important for the action recognition task. It is necessary to memorize this periodic information. Moreover, there always exists redundant information in an action sequence. That is to say, not every frame in a video sequence is important for action recognition. Thus, how to select key frames and extract discriminative features also needs to be carefully considered.

In this paper, we propose an end-to-end network architecture, termed as Adaptive Attention Memory Graph Convolutional Networks (AAM-GCN) for human skeleton action recognition. It employs graph convolution to adaptively construct the spatial configuration in one frame and uses multiple bidirectional GRU layers to extract temporal information. Concretely, we construct an adaptive spatial graph convolution (ASGC) module to explore the spatial configuration feature. When constructing the spatial graph, an adaptive matrix is designed that could infer relations between any joints in a data-driven manner without weakening the relation of physically connected joints according to the actions. Then, the topology of the constructed graph is dynamically established during the training process. The attention memory module is implemented via multi-bidirectional GRU layers to build an attention-enhanced memory. It could remember the long-range temporal context before and after the actions. In addition, not all frames in the action sequence are informative, and there are lots of redundancies. The key frames carrying more discriminative information for action recognition should be enhanced during training. Therefore, we incorporate attention mechanisms to the memory for selecting the important frames. Moreover, the attention-weighted memory is refined progressively by multiple iterations for recognition.

The advantage of our proposed method could be described as: (1) The constructed adaptive graph can effectively capture the latent dependencies between arbitrary joints, including the ones which do not have a physical connection but have strong correlations in the actions, which is more suitable for real actions that need the collaboration of different body parts. (2) The memory model is capable of modeling long-range temporal relationships over distant frames, which is beneficial for extracting discriminative features because it can provide a temporal context for the actions. (3) Due to the forward and backward directions, the bidirectional GRU could encode both what happened in the past and what will happen in the future, which can eliminate ambiguities of the actions, such as “put on hat” and “take off hat” via long time observation. (4) The attention could help the model to select key frames in the action sequence.

The main contributions of our work are summarized as follows:

- We propose an AAM-GCN network to model dynamic skeletons for action recognition, which can construct the graph structure adaptively during the training process and explicitly explore the latent dependency among the joints.
- By constructing an attention-enhanced memory, AAM-GCN can selectively focus on key frames and capture both long-range discriminative temporal features in the past and the future.
- We evaluate the proposed model on three large datasets: NTU RGB+D [28], Kinetics [29] and Motion Capture Dataset HDM05 [30]. It exhibits superior performance than some other state-of-the-art methods in both constrained and unconstrained environments. Furthermore, we conduct an ablation study to demonstrate the effectiveness of each individual part of our model.

## 2. Related Works

The human skeleton can effectively represent the human body structure and movement due to the fact that it is robust to dynamic circumstances and irrelevant to appearance. For action recognition tasks, early works [14,16] often used handcrafted features to design the model. In recent years, deep learning has received more attention, many Convolutional Neural Network (CNNs) and Recurrent Neural Network (RNNs)-based methods have been proposed for skeleton action recognition [13,31,32]. The models based on CNN usually consider the joint coordinates as a grid structure and implement convolution to extract discriminative features [13,33,34,35,36]. Xie et al. [13] proposed a memory attention network that took each element in the coordinate vector of all the joints as 2D grid data and then conducted convolution on it. Li et al. [33] designed a hierarchical structure to extract co-occurrence features of skeleton joints using CNN.

Compared with CNN, RNN has a stronger ability to model sequence data. Various RNN-based models have been applied to the action recognition field [37]. Models based on RNN usually use the coordinates of joints and consider the skeleton data as a sequence of vectors along a temporal dimension [6,11,28,38,39]. As a variant of RNN, Long Short-Term Memory (LSTM) has also been widely employed to learn the temporal dynamics of skeleton sequences [12,18,40]. Liu et al. [12] arranged the joints as a chain along the spatial dimension and then employed the coordinates with LSTM to build a global context memory for action recognition. Du et al. [11] constructed a part-based hierarchical RNN architecture in which the skeleton was divided into several parts and then fed to a hierarchical Recurrent Neural Network. Song et al. [38] adopted an attention mechanism to select key joints and focused on discriminative features via LSTM. However, due to the fully connection structure within it, the LSTM-based networks suffered from high computation complexity problems. Consequently, the Gated Recurrent Unit (GRU), a simplified variant of LSTM, was proposed by Cho et al. [41], which is efficient and computationally inexpensive.

Although the existing CNN and RNN-based methods have achieved remarkable results, there still exist limitations for using CNNs and RNNs to model the skeleton data. First, CNN and RNN cannot exactly describe the spatial-temporal structure of the body. Second, the aforementioned CNN and RNN methods all characterize the topology of the human skeleton joints as pseudo images or vector sequences rather than a graph. Thus, the spatial structure of the body skeleton is neglected in them.

Graph Convolutional Neural Networks (GCN) have been widely used in many areas, such as social networks and biological data analysis [2]. Due to the topology of the human skeleton structure, it can be naturally represented by a graph; the graph convolution has been applied to a skeleton action recognition task recently. Yan et al. [20] first applied GCN to the skeleton data for human action recognition. After that, a series of GCN-based methods have emerged. Shi et al. [19] proposed a Two-stream Adaptive Graph Convolutional Network (2s-AGCN). They used both joints and bones to build a two-stream work separately and fused the sores for final recognition. Li et al. [17] introduced an Actional-Structural Graph Convolutional Networks (AS-GCN) and designed two types of links: actional link and structural link for the human skeleton. Combining GCN and LSTM, Si et al. [18] presented an attention human action recognition model based on hierarchical spatial reasoning and temporal stack learning network.

More recently, Liu et al. [42] proposed a feature extractor (MS-G3D) that modeled cross space-time joint dependencies. However, only the neighbor nodes in a few adjacent frames will be convoluted for the central node. The information in the temporal field is relatively local. Dynamic GCN [43] is a hybrid GCN-CNN framework. They designed a dynamic graph topology for different input samples as well as graph convolutional layers of various depths. Nevertheless, the generality of the graph structure and the collaboration of joints are still to be considered. Plizzari et al. [44] proposed a Spatial-Temporal Transformer network (ST-TR), which modeled dependencies between joints using the transformer self-attention operator. It combined the Spatial Self-Attention module and the Temporal Self-Attention module in a two-stream way; they got better results than models that used both joint and bone information as the input data. Aside from that, the input data in ST-TR were pre-processed by [25]. Chen et al. [45] proposed a Channel-wise Topology Refinement Graph Convolution (CTR-GC) to dynamically learn different topologies and effectively aggregate joint features in different channels. They employed four modalities’ data (joint, bone, joint motion and bone motion) as inputs. On the other hand, the input data of CTR-GC and ST-TR are both pre-processed before training. During the training process, CTR-GC adopted a warm-up strategy at the beginning of the training process.

Although the previous methods have remarkable performance, how to construct a specific graph to effectively represent the human skeleton data and extract discriminate features are still challenging issues. 

The attention mechanism has received extensive attention in many applications [46,47]. It can help the network to focus on key information while neglecting the inessential information. Xu et al. [48] employed an attention mechanism in their image caption generation work. Yao et al. [49] applied attention to the temporal dimension for their video captioning task. Stollenga et al. [50] adopted an attention mechanism to the image classification field. Luong et al. [47] combined global attention and local attention to computing different attention weights for neural machine translation. Some deep learning-based works have adopted an attention mechanism in action recognition [46,51]. The hidden information of the previous time step in LSTM is often used for calculating attention. Liu et al. [12] proposed a global context-aware attention LSTM network (GCA-LSTM) for skeleton action recognition. They introduced an iterative attention mechanism using global context information to choose the informative joints in an action video clip. Similarly, Song et al. [38] computed attention weights to focus on key frames and more informative joints. Xie et al. [13] designed a temporal attention recalibration module to weigh each frame in the skeleton action sequence. Si et al. [18] proposed an attention-enhanced graph convolutional LSTM network that incorporated soft attention to automatically measure the importance of each joint and adaptively selected the key ones.

## 3. Graph Convolutional Networks

**Figure 1:**
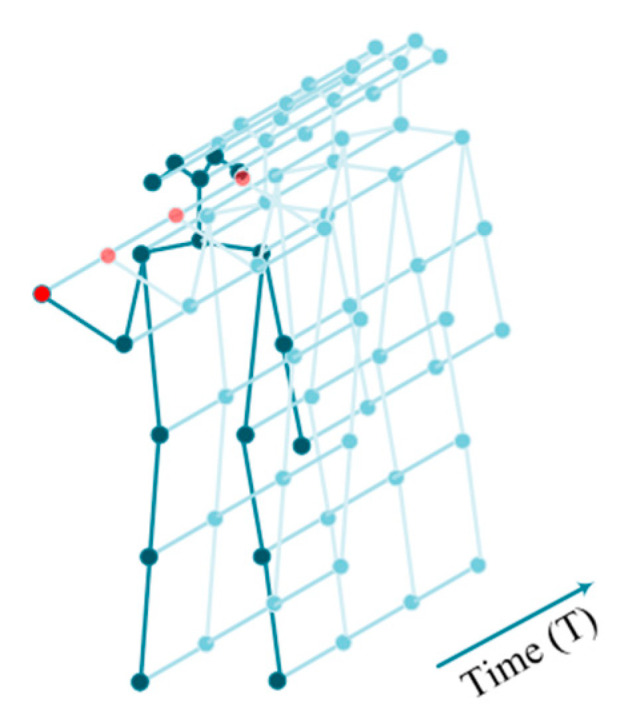
Illustration of spatial-temporal graph of an action clip with multiple frames.

The skeleton data can be acquired by depth sensors or generated by pose estimation algorithms from a raw RGB video clip. The position of each joint is represented as a 2D or 3D coordinate vector. As a result, the skeleton in a frame is represented as a set of joint coordinates. In GCN-based methods [18,20], the human skeleton dynamics in an action video clip are often modeled as a spatial-temporal graph. In the graph, the joints are considered as nodes, and physical connections between joints in a frame are considered as spatial edges. Additionally, temporal edges are added to connect the same joint between consecutive frames. The joint coordinates are considered as the attribute of the corresponding node. We follow [18,20] as our basic approach for constructing the graph. The detailed process is described below:

In GCN-based action recognition works [18,20], the dynamics of the human skeleton sequence for performing actions with N joints and T frames are denoted as a spatial-temporal graph G=(V, E). The node set $V=\left\{v_{ti}|t=1,\;\ldots ,\;T;i=1,\;\ldots ,\;N\;\right\}$ contains all the joints in the skeleton sequence. The edge set E contains two subsets: the intra-frame edge set $E_{s}$ and the inter-frame edge set $E_{F}$. For clear demonstration, an example of a spatial-temporal graph is illustrated in Figure 1. An action clip is composed with T frames, and the person in each frame has N joints, and each joint is represented by 2D or 3D coordinates. Thus, the action clip can be represented by the joint-coordinates vector $X\in {\mathbb{R}}^{N\times T\times d}$, where d denotes the dimension of coordinates. 

For the arbitrary node $v_{ti}$ in V, its neighbor set in the current frame is defined as $N\left(v_{ti}\right)=\{v_{tj}|d\left(v_{ti},v_{tj}\right)\leq D\}$, where $d\left(v_{ti},v_{tj}\right)$ is the minimum path length from $v_{tj}$ to $v_{ti}$ and D is set to 1. According to the strategies designed for partitioning the neighbor set [20], different labeling functions $f:V_{t}\to \left\{1,\;2,\;\ldots ,\;M\right\}$ are designed to partition the neighbor set $N\left(v_{ti}\right)$ of node $v_{ti}$ into M subsets at the $t^{th}$ frame. Each node in the neighbor set is assigned a label from {1, 2, …, M}. The nodes in the same subset have the same label and share the convolution weights, and then the graph convolution is conducted as:

(1) $$g_{conv}\left(v_{ti}\right)=\sum_{v_{tj}\in N(v_{ti})}\frac{1}{Q_{ti}\left(v_{tj}\right)}X\left(v_{tj}\right)W(f\left(v_{tj})\right)$$

where $X\left(v_{tj}\right)$ represents the feature of neighbor node $v_{tj}$. W(·) is a weight function, which allocates weights according to different labels. The label of each node $v_{tj}$ that in neighbor set $N\left(v_{ti}\right)$ is computed by $f\left(v_{tj}\right)$, according to the partitioning strategy. The neighbor nodes that have the same label will be allocated the same weights. $Q_{ti}\left(v_{tj}\right)$ is the number of nodes of each subset according to the label, which is considered as the normalizing term and $g_{conv}\left(v_{ti}\right)$ represents the result of graph convolution computation at node $v_{ti}$. The graph convolution could be implemented as in [21]:

(2) $$g_{conv}{=\Lambda }^{-\frac{1}{2}}{(A+I)\Lambda }^{-\frac{1}{2}}\mathrm{XW}$$

where Λ is a degree matrix and the diagonal element $\Lambda_{ii}=\underset{j}{\sum }{(A}_{ij}{+I}_{ij})\;$, A is the adjacency matrix for encoding the connections of nodes before partitioning the neighbor sets and I is the identity matrix representing self-connections. The topology of a human skeleton graph in a frame is described by A and I. According to the partitioning strategy, the labeling function divides each neighbor set into M subsets. Therefore, the adjacency matrix can be dismantled as $A+I=\overset{M}{\underset{m=1}{\sum }}A_{m}\;$, m∈{1, 2, …, M}, where $A_{m}$ represents the adjacency matrix of each subset and m is the label. $A_{m}$ has the same size as the original N×N normalized adjacency matrix, N is the number of joints. Given $A_{m}$, Equation (2) can be represented as:

(3) $$g_{conv}=\sum_{m=1}^{M}\Lambda_{m}^{-\frac{1}{2}}A_{m}\Lambda_{m}^{-\frac{1}{2}}{\mathrm{XW}}_{m}$$

**Figure 2:**
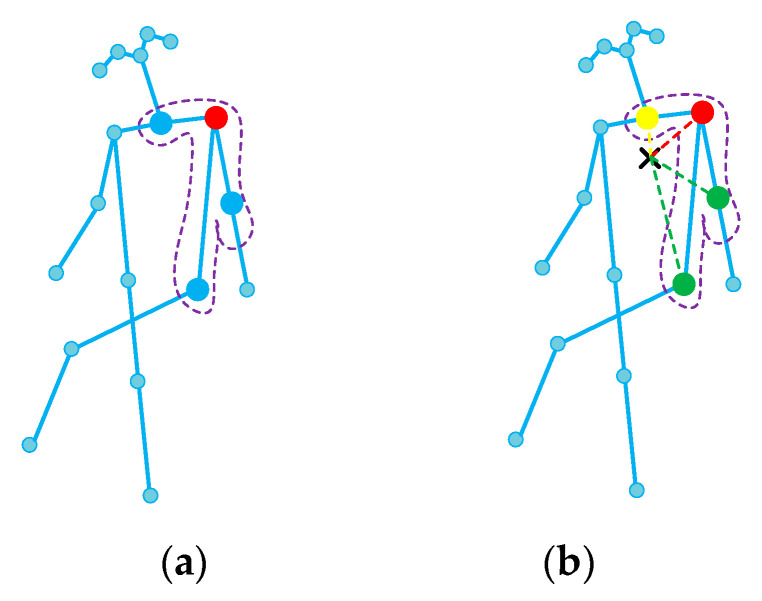
The spatial configuration partitioning strategy for partitioning the neighbor set. (**a**) Input skeleton of a person in a frame. The neighbor nodes and their root node (colored in red) are circled with a dashed line. (**b**) The nodes are labeled according to their distances from the skeleton gravity center (black cross) compared with that of the root node. The yellow ones represent centripetal nodes that have a shorter distance. The green ones represent centrifugal nodes that have a longer distance than the root node.

Taking the spatial configuration partitioning, for instance, see Figure 2, the neighbor set of the root node is partitioned into M=3 subsets: (1) the root node itself; (2) the centrifugal group: the neighbor nodes that are further to the gravity center of the skeleton than the root node; or (3) the centripetal group. Therefore, the dismantled adjacent matrix $A_{m}$ represents correlations of nodes in each subset.

For extracting temporal information, Yan et al. [20] simply conducted temporal convolution along the temporal dimension with a kernel size of nine. Nevertheless, it ignored the correlation of temporal context and could not focus on discriminate information.

## 4. Adaptive Attention Memory Graph Convolutional Network

**Figure 3:**
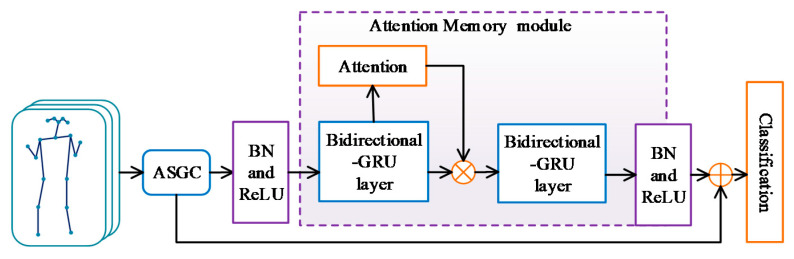
The overall architecture of the proposed Adaptive Attention Memory Graph Convolutional Networks.

In view of the disadvantages of previously analyzed methods above, we propose a novel Adaptive Attention Memory Graph Convolutional Network (AAM-GCN). In this section, we will introduce the individual component of our model in detail. The overall architecture of AAM-GCN is shown in Figure 3. The skeleton action sequence is represented as a multi-frame sequence of the joint-coordinates vector and fed to the adaptive spatial graph convolution (ASGC) module to extract the spatial feature. After Batch Normalization (BN) and ReLU, the spatial feature will be sent to the attention memory module to obtain the attention-enhanced temporal feature. To extract effective temporal information without weakening the spatial configuration information, we concatenate the spatial and temporal features as the final discriminative feature for action recognition. More specifically, the AAM-GCN not only uses the temporal attention-enhanced memory information but also delivers the spatial configuration information of ASGC by the identity shortcut.

### 4.1. Adaptive Spatial Graph Convolution

The adaptive spatial graph convolution (ASGC) is designed to extract the spatial information of the human skeleton sequence adaptively. The spatial-temporal graph is often designed to describe the structure of the human body and the dynamics of actions. The spatial graph structure [18,20] introduced in Section 3 is predefined and set manually, which will be fixed during the training process. The adjacency matrix A encodes the connections of nodes and decides the topology of the spatial graph according to the partitioning strategy. However, it can only capture relations between the joints connected by physical connections [20]. If the element $A_{ij}=0$ in A, it means that joint $v_{ti}$ and $v_{tj}$ will never establish correlation in the action representation. This may not be suitable for real actions that happen with the collaboration of arbitrary joints. For example, the two hands have strong correlations when clapping. The hand and head will move collaboratively when brushing teeth and talking on the phone. In fact, these joints are not directly connected by physical connections but have strong correlations. 

To solve this problem and construct a more generalized skeleton graph, we design an adaptive data-driven graph structure to capture rich dependencies among arbitrary joints. That is, we add an adaptive matrix P to the original adjacency matrix A for getting an adaptive adjacency matrix $A^{a}=A+P$ without weakening correlations of physical connecting joints. Consequently, the original item $A_{m}$ in Equation (3) will be changed by $A_{m}^{a}=A_{m}+P_{m\;}$, and Equation (3) will be modified as:

(4) $$g_{conv}=\sum_{m=1}^{M}\Lambda_{m}^{-\frac{1}{2}}A_{m}^{a}\Lambda_{m}^{-\frac{1}{2}}{\mathrm{XW}}_{m}=\sum_{m=1}^{M}\Lambda_{m}^{-\frac{1}{2}}\left(A_{m}+P_{m\;}\right)\Lambda_{m}^{-\frac{1}{2}}{\mathrm{XW}}_{m}\;$$

where $P_{m\;}$ is a learnable parameter matrix with initial value of all zeros; it has the same size of $A_{m\;}$. In contrast to $A_{m\;}$, the elements in $P_{m\;}$ are parameterized and will be optimized according to the training data. During the training process,${\;P}_{m\;}$ will update its elements according to different actions and capture the correlations between any joints. The graph learns according to the training data in a data-driven manner. The elements in $A_{m}^{a}$ not only represent the connection between each pair of joints, but also indicate the intensity of the correlation. 

As a result, the adaptive graph adjacent matrix $A_{m}^{a}$ could establish correlations between arbitrary joints performing collaboratively in actions. It is more flexible than the predefined fixed one.

To eliminate inter-frame redundancy and increase the temporal receptive field, we conduct a temporal average pooling operation. It can not only improve the perception ability of the module but also reduce the computation cost. After temporal pooling, we obtain a multi-frame clip feature instead of a single frame, which will be more effective for perceiving temporal dynamics.

In the computation process, the input skeleton data of the ASGC module is a 2D or 3D joint-coordinate vector sequence represented by $X_{0}\in {\mathbb{R}}^{C_{0}\times T_{0}\times N}$, where $C_{0}$ is the input coordinate dimension, $T_{0}$ is the number of frames and N is the number of joints. After spatial graph convolution and temporal pooling, we can acquire the spatial configuration feature $X_{s}\in {\mathbb{R}}^{C\times T\times N}$, where C is the number of channels and T is the temporal dimension after ASGC. The acquired spatial feature will be fed into the attention memory module to learn the temporal relations. The attention memory module will be introduced in the next section.

### 4.2. Attention Memory Module

The attention memory module is implemented via multi-bidirectional GRU layers to establish an attention-enhanced memory. It can effectively capture the temporal dynamics of the human skeleton action sequence. Due to the forward and backward directions, bidirectional GRU can not only remember what happened in the past but also employ the information in the future. In addition, not all frames in the action sequence are informative and there are lots of redundancies. The key frames carrying more discriminative information for action recognition should be enhanced during training. Therefore, we incorporate the attention mechanism to the memory for choosing the important frames.

**Figure 4:**
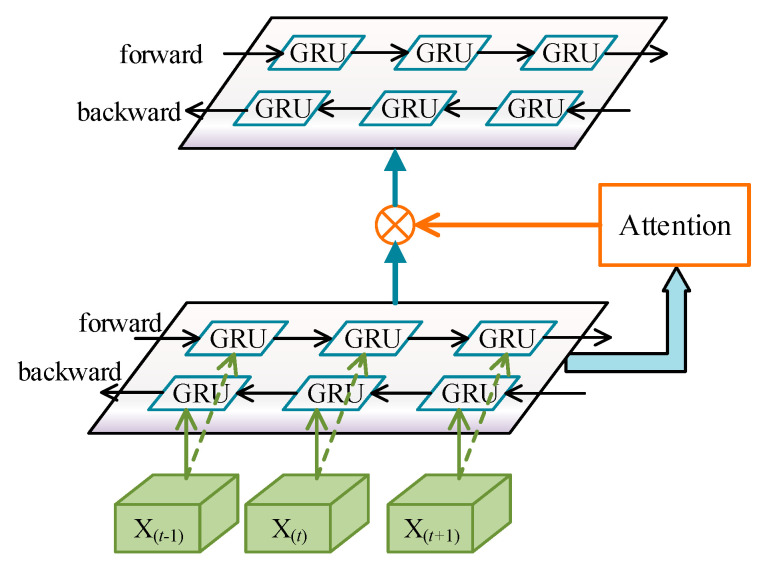
The structure of the attention memory module.

The structure of the attention memory module is shown in Figure 4, which contains two bidirectional GRU layers. The first layer encodes the spatial feature produced by ASGC and generates the initial memory. Then this initial memory matrix is weighted by the attention procedure. Since the attention-enhanced memory is achieved, we feed it to the second layer to refine the memory. We propose a recurrent mechanism to optimize the memory. Multiple iterations can be carried out to refine the memory progressively. Finally, the output of the last bidirectional GRU layer combined with the spatial structure acquired in ASGC will be concatenated to predict the action label. The detailed process is described below: 

After the spatial graph convolution operation that has been introduced in Section 4.1, we get the spatial feature matrix $X_{s}\in {\mathbb{R}}^{C\times T\times N}$. Then, it will be fed into the attention memory module to learn the temporal features. The attention memory module is composed of multi-bidirectional GRU layers. The number of hidden neurons in each GRU is denoted by K. The intermediate hidden states of the forward and backward GRU are full of spatial and temporal information, which is useful for selecting the key frames. In particular, we adopt a soft attention mechanism to adaptively focus on the key frames that are beneficial for action recognition.

Let $H^{\left(n\right)}\in {\mathbb{R}}^{T\times 2K}$ denote the output hidden state of the ${\left(n+1\right)}^{th}$ bidirectional GRU layer. For each time step, we aggregate the row of $H^{\left(n\right)}$ and generate a query vector $Q^{\left(n\right)}\in {\mathbb{R}}^{T\times 1}$ as:

(5) $$Q^{\left(n\right)}={\left[q_{0},\ldots ,q_{t},\ldots ,q_{T-1}\right]}^{T},\;t=0,\;1,\;\ldots ,\;\left(T-1\right);\;n\geq 0$$

where $q_{t}=relu\left(\frac{1}{2K}\sum_{i=0}^{2K-1}H_{ti}^{\left(n\right)}\right)$. The attention score $F^{\left(n\right)}\in {\mathbb{R}}^{T\times 2K}$ of the ${\left(n+1\right)}^{th}$ layer will be calculated as:

(6) $$F^{\left(n\right)}=sigmoid\left(W_{0}^{\left(n\right)}tanh\left(W_{1}^{\left(n\right)}H^{\left(n\right)}+W_{2}^{\left(n\right)}Q^{\left(n\right)}+b_{1}^{\left(n\right)}\right)+b_{0}^{\left(n\right)}\right)$$

where $W_{0}^{\left(n\right)},\;W_{1}^{\left(n\right)},\;W_{2}^{\left(n\right)}$ are learnable parameter matrices and $b_{0}^{\left(n\right)},\;b_{1}^{\left(n\right)}$ are the bias.

The memory $ℳ\in {\mathbb{R}}^{T\times 2K}$ can be computed from the hidden states of GRU from two different directions. At first, the initial memory ${ℳ}^{\left(0\right)}\in {\mathbb{R}}^{T\times 2K}$ in the first layer is computed as:

(7) $${ℳ}^{\left(0\right)}=H^{\left(0\right)}=G_{F}\left(X_{s}\right)⊕G_{B}\left(X_{s}\right)$$

where $G_{F}\left(\cdot \right)$ and $G_{B}\left(\cdot \right)$ represent the computation of hidden states by forward and backward GRU, respectively. We concatenate the output hidden state of each direction to construct the initial memory matrix ${ℳ}^{\left(0\right)}$ in Equation (7), ⊕ denotes the concatenating operation. 

Let ${ℳ}^{\left(n\right)}\in {\mathbb{R}}^{T\times 2K}$ denote the memory of the ${\left(n+1\right)}^{th}$ bidirectional GRU layer. After the attention matrix $F^{\left(n\right)}$ is achieved in Equation (6), the attention-enhanced memory ${ℳ}_{F}^{\left(n\right)}$ would be calculated as:

(8) $${ℳ}_{F}^{\left(n\right)}=F^{\left(n\right)}⊗{ℳ}^{\left(n\right)}$$

where ⊗ denotes the element-wise multiplication.$\;{ℳ}^{\left(n\right)}$ and $F^{\left(n\right)}$ in attention memory module could be jointly learned during training. 

To get a more reliable memory, we propose a recurrent mechanism to iteratively refine the memory. The attention-enhanced memory ${ℳ}_{F}^{\left(n\right)}$ of the current layer will be fed into the next bidirectional GRU layer to refine the memory iteratively:

(9) $${ℳ}^{\left(n+1\right)}=H^{\left(n+1\right)}=G_{F}\left({ℳ}_{F}^{\left(n\right)}\right)⊕G_{B}\left({ℳ}_{F}^{\left(n\right)}\right)\;n\geq 0$$

where ${ℳ}^{\left(n+1\right)}$ represents the refined memory computed by the next layer. After N iterations, the final memory of skeleton joints ${ℳ}^{\left(N\right)}\in {\mathbb{R}}^{T\times 2K}$ is achieved. For simplicity, we use ℳ instead of ${ℳ}^{\left(N\right)}$ to represent the final memory. Note that, the output memory of the last layer aggregates all node features for classification. Consequently, the multi-bidirectional GRU layers extract the temporal information from the spatial feature of skeleton and summarize memory ℳ across the input sequence. 

To extract the temporal information of key frames without weakening the information of the spatial structure, the spatial features produced by ASGC are delivered by the identity shortcut to concatenate with temporal features. Finally, the concatenated features are used for action recognition. 

### 4.3. Model Architecture and Training Detail

To integrally capture temporal features and the spatial relationships among arbitrary joints, we combine the adaptive spatial graph convolution (ASGC) and attention memory module to develop the Adaptive Attention Memory Graph Convolutional Network (AAM-GCN) for skeleton action recognition. 

We first normalized the input data via a batch normalization (BN) layer at the beginning and then adopted ASGC to obtain the spatial features of joints. The ASGC module is composed of nine graph convolution blocks; the feature dimensions are 64, 128 and 256 in the first, second and last three blocks. In each block, the graph convolution layer is followed by a BN and a non-linear activation ReLU layer. We also add a temporal pooling operation to improve the efficiency after the convolution layer. To avoid overfitting, we set a dropout layer with a probability of 0.5 in each block. For the neighbor selection method in ASGC, the neighbor nodes connected directly with the root node (D=1) will constitute the neighbor set. Similarly, the spatial configuration partitioning strategy is adopted as the graph labeling function as [20]. Therefore, the neighbor set will be partitioned into M=3 subsets: the centrifugal group, the centripetal group and the root node itself. The number of hidden neurons in the first and second bidirectional layers is 128 and 64, respectively. Extensive experiments are conducted on PyTorch1.2.0 using one GTX-1080 GPU. The model is trained for 50 epochs for each dataset. The batch size is 32. Stochastic Gradient Descent (SGD) is used as the optimization function, and the initial learning rate is 0.01. It is divided by 10 after every 10 epochs. To avoid overfitting, we adopt the data-augmentation method as ST-GCN [20]. Cross-entropy [52] is chosen as the loss function.

## 5. Experiments

To evaluate the effectiveness and generality of the proposed AAM-GCN model, experiments are conducted on both constrained and unconstrained action recognition datasets: NTU RGB+D [28] and Kinetics [29]. We also evaluate our model on Motion Capture Dataset HDM05 [30]. First, we compare our model with other state-of-the-art methods for exhibiting the superior performance of the proposed model. Then, exhaustive ablation studies are conducted on the two large datasets to examine the importance and effectiveness of each individual part of AAM-GCN.

### 5.1. Datasets

**Figure 5:**
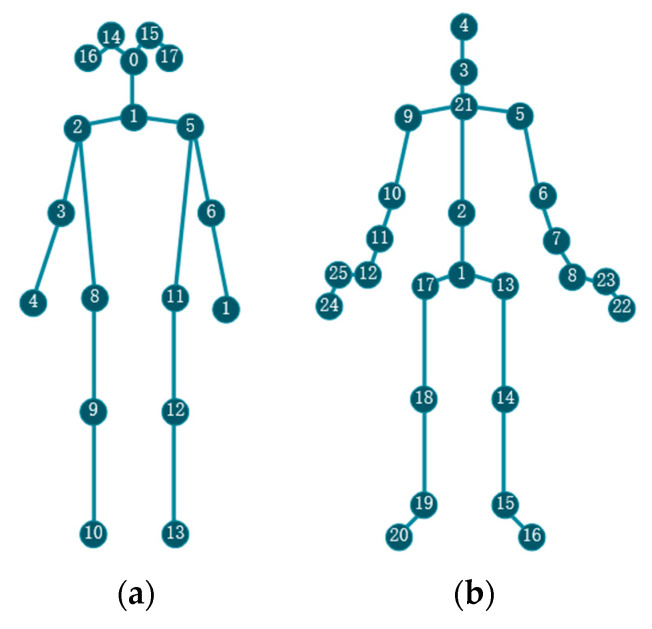
The skeleton structure in Kinetics and NTU RGB+D datasets. (**a**) The joint label and connection of Kinetics. (**b**) The joint label and connection of NTU RGB+D.

**Kinetics:** Kinetics [29] is a large unconstrained action recognition dataset that collects videos from YouTube. It contains approximately 300,000 video clips and has a great variety covering 400 human action classes. Each action class includes more than 400 action clips. Each clip lasts around 10s and is assigned an action label. The actions are human-focused and cover a broad range of classes, including human–object interactions, such as playing instruments, as well as human–human interactions, such as shaking hands. The ground truth annotations are provided for each clip, which means each clip is assigned an action label. The dataset is divided into a training set of 240,000 video clips and a validation set of 20,000. The original videos provided by Kinetics are raw RGB videos without skeletons. Yan et al. [20] extracted the location information of skeleton joints of each frame by pose estimation algorithm OpenPose [9]. The original videos were first resized to 340 × 256 and then processed by the OpenPose toolbox to get the 2D joint coordinates (X, Y) and confidence scores C for 18 joints. The attribute of each joint was represented as a vector ${\left(X,\;Y,\;C\right)}^{T}$. As a result, the skeleton of a person was represented by a sequence of 18 vectors. The skeleton structure in Kinetics is shown in Figure 5a. For multi-person video clips, the two people with the highest average joint confidence score were chosen. For easy computation, the clip was padded by replaying the start to make each sample have the same length with 300 frames. The skeleton data of the Kinetics dataset was released by [20], we use it to evaluate our model. Because of the great variety and unconstrained condition of the dataset, it is much harder to perform the action recognition task on it, so we report top-1 and top-5 accuracies in the validation set as in [20]. In the case of the top-1 accuracy, it needs to check whether or not the top class (the one with the highest probability) in the prediction result is the same as the target label; in the case of the top-5 accuracy, it needs to check whether or not the target label is in the top 5 predictions (the five with the highest probabilities). In both cases, the top accuracy is computed as the number of times a predicted label matched the target label, divided by the number of data points evaluated. 

**NTU RGB+D:** NTU RGB+D [28] is a large, constrained action recognition dataset under a lab environment, which contains 56,000 action clips with 60 classes. The clips in this dataset include daily, mutual and health-related actions. Ground truth labeling is assigned for each clip. The actions are performed by 40 volunteers and captured by three Microsoft Kinect v2 depth sensors simultaneously. The cameras record actions from different horizontal angles and provide human joint skeleton 3D spatial coordinates (X, Y, Z). The dataset provides 25 joint-location information for each person, and there are no more than two people in each video clip. The skeleton structure and joint labels in NTU RGB+D are shown in Figure 5b. The original work [28] recommends two benchmarks for evaluating models: Cross-Subject and Cross-View. Cross-Subject evaluation contains 40,320 clips for training and left 16,560 for validation. The subjects in the training set are different from the validation set. Cross-View evaluation contains 37,920 clips for training and 18,960 for validation. The training clips are captured by camera two and camera three, and the evaluation clips are captured by camera one. All the cameras record the same subject with different perspectives. We evaluate our model following [28] and report top-1 accuracy on both benchmarks.

**HDM05:** HDM05 [30] is a motion capture dataset performed by five non-professional actors. It is captured by an optical marker-based technology and contains 2337 skeleton sequences for 130 actions. Each action clip contains multi-frames and is assigned an action label. It provides information on 31 joints for the actor in each frame. We process the data following the protocols in [53]. As stated in [53], some action classes in the 130 actions should be merged into the same category, e.g., walk two steps and walk four steps could be merged into a single action “walk”. Jogging starting from the air and jogging starting from the floor could be considered as the same “jogging” action. After the sample combination, the actions are reduced to 65 categories.

### 5.2. Comparisons with the State-of-the-Art

In this section we compare the performance of our proposed model with some state-of-the-art methods. Table 1 shows the action recognition accuracy of top-1 and top-5 of various methods on Kinetics dataset. Deep LSTM [28] is an RNN-based method, and Temporal Conv [34] is a CNN-based method. The spatial configuration information of human skeletons, which is important for action recognition, was ignored in these methods. ST-GCN [20], AS-GCN [17] and ST-GGN [54] are GCN-based models. The human skeleton graph in ST-GCN is fixed, which can not exactly describe various actions. AS-GCN simply conducted convolution along a temporal dimension and could not focus on key frames. ST-GGN failed in establishing relation context in the temporal files. From Table 1 we can see that the proposed AAM-GCN outperforms Deep LSTM, Temporal Conv, ST-GCN and ST-GGN. 

Although AS-GCN gains better performance than ours, the model is much more complicated. The total parameters of AS-GCN are 7.41 M compared with the 3.76 M of ours, nearly twice as much as ours. The floating point of operations (FLOPs) of our model is 19.43 G compared with 35.53 G of AS-GCN, which indicates that the complexity of our model is much lower. Aside from that, the AS-GCN model is trained for 100 epochs; however, our model is only trained for 50 epochs, and the convergence speed is faster. All of this proves that AAM-GCN is effective and much faster.

We also adopt another pose estimation algorithm DeepCut [55], for extracting skeleton sequences from the Kinects dataset. DeepCut is a CNN-based method that could estimate the location of human joints and provide the coordinates of them. We employ the skeleton data as the input and get similar results on Kinetics. The results are shown in Table 1 as AAM-GCN (DeepCut).

**Table 1:** The action recognition accuracy of top-1 and top-5 of various methods on the Kinetics dataset.

Methods	Top-1 Acc (%)	Top-5 Acc (%)
Deep LSTM [28]	16.40	35.30
Temporal Conv [34]	20.30	40.00
ST-GCN [20]	30.70	53.80
ST-GGN [54]	33.10	55.20
AS-GCN [17]	34.80	56.50
AAM-GCN (DeepCut)	33.24	55.58
AAM-GCN (ours)	33.12	55.65

The proposed model is trained on two protocols of the NTU RGB+D dataset separately. The results are compared with other state-of-the-art methods in Table 2. The methods in [6,12,15,39] are based on RNN, while the other two methods in [35,36] are based on CNN. The methods in [20,56] are GCN-based. ST-LSTM [6] is a spatio-temporal skeleton action recognition method and performs action classification on a local representation. Two-stream RNN [15] adopted two parallel RNNs to extract spatial and temporal features separately. GCA-LSTM [12] combined LSTM with global attention information to focus on key joints. These methods employed the coordinates of joints and arranged them as a chain when extracting spatial features. Therefore, the spatial relations of joints and body structure are ignored. DS-LSTM [57] trained a three-layer denoising sparse LSTM structure to capture the temporal connections in the skeleton sequence. Nevertheless, only the information of the previous time step was used with LSTM. It could not take advantage of the information in the future, which is beneficial for recognition. ST-GCN [20] used graph convolution to construct spatial configuration and temporal convolution to extract temporal information. The graph structure designed for convolution had limitations for representing the real actions; because it cannot establish relations between joints that are not physically connected. The relationship between skeleton nodes and extracted multiple skeleton features for extracting spatial features was increased by 3D-GCN [56]. However, the frames were treated equally when extracting temporal features. It is not suitable and inefficient since there are lots of redundancies in the frames. Compared with the RNN-based method [39], AAM-GCN outperforms 3.3% and 2.5% in terms of Cross-Subject and Cross-View. Compared with the CNN-based method [36], our proposed model improves the accuracy by 3.2% and 5.3%, respectively. Moreover, the accuracy of our model is higher than ST-GCN [20] by 1.2% and 1.8% on the two benchmarks. The results in Table 2 demonstrate that our model is much superior to other state-of-the-art methods.

HDM05 is a motion capture dataset utilized on skeleton-based works. Compared with other large-scale datasets that have emerged in recent years, it is much smaller. Therefore, there are much fewer action recognition networks conducting an evaluation on it. We evaluate our method on it and compared it with some other state-of-the-art methods. The evaluation results are shown in Table 3. From Table 3, we can see that our proposed network yields better results than other skeleton-based action recognition methods. Multi-layer Perceptron networks are designed to classify each frame [53]; however, they could not establish temporal corresponding effectively. Furthermore, the spatial structure of the human body is not considered in this method. Hierarchical RNN [11] is a deep Recurrent Neural Network architecture with handcrafted subnets utilized for skeleton-based action recognition. The handcrafted hierarchical subnets and their fusion ignore the inherent correlation of joints. Zhu et al. [40] employed LSTM and introduced a regularization scheme to learn the co-occurrence features of skeleton joints. Nevertheless, it could only employ the information in the past tense, and the important future information is not considered in their method.

### 5.3. Visualization of the Actions

**Figure 6:**
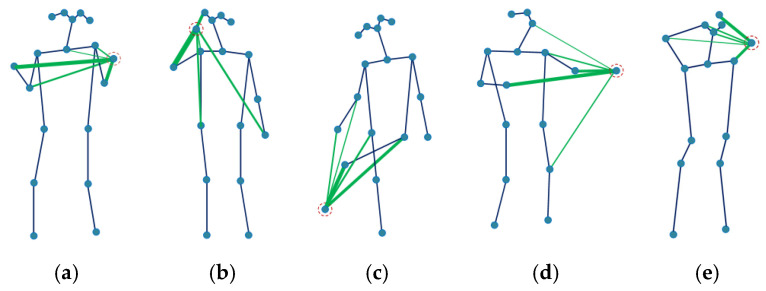
Visualization of relations between the specific joint that circled with dash line and other joints with AAM-GCN. (**a**) clapping; (**b**) talking on the phone; (**c**) kicking; (**d**) beating; (**e**) comb hair.

**Figure 7:**
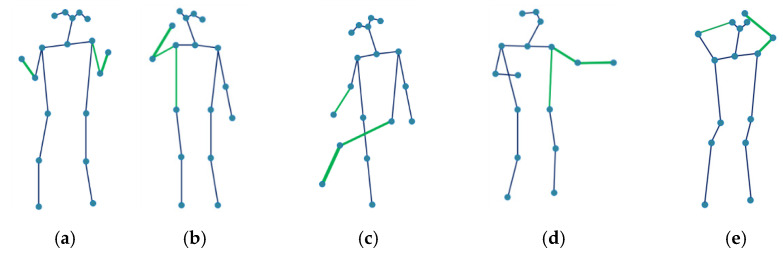
Visualization of the most informative joints with ST-GCN. (**a**) clapping; (**b**) talking on the phone; (**c**) kicking; (**d**) beating; (**e**) comb hair.

The joints move collaboratively when performing actions; different actions may activate different body parts. For example, the two hands have strong correlations with each other when clapping. The foot interacts with the arm and leg when kicking something. Figure 6 shows the inferred relations between the specific node, which is circled with a dashed line, and other joints by AAM-GCN. The green lines represent the first five strongest relations of the corresponding joint. The wider lines represent stronger relations. In Figure 6a,b,d,e, clapping, talking on the phone, beating and combing hair are mainly upper limb actions; kicking in Figure 6c is mainly a lower limb action. The circled joint in each action interacts not only with physically connected joints but also with other parts of the body. In order to make a comparison, we also visualize the same actions with ST-GCN; the results are shown in Figure 7. The green lines show the connection between the most informative joints and its neighbors in action. From Figure 7 we can see that ST-GCN could only establish a correlation between the joints connected by physical connection. It is not suitable because of that; joints often move collaboratively in real actions even if they are not physically connected. The visualization shows that our model can more effectively capture relations among any joints than other methods.

### 5.4. Ablation Study

To analyze and examine the importance and effectiveness of each individual part of the proposed Adaptive Attention Memory Graph Convolutional Networks (AAM-GCN), we conduct ablation experiments on the NTU RGB+D [28] and Kinetics [29] datasets in this section.

#### 5.4.1. Effect of Adaptive Graph Structure

Because of the fact that our work is based on ST-GCN [20], it is used as a baseline work to compare with the ablation experiments. To analyze the effect of the adaptive graph structure of our model, the original graph adjacency matrix as in [20] is used without adaptive structure. Spatial configuration partitioning is adopted as the partitioning strategy, as in [20]. This degraded method is termed as AAM-GCN wo/adap. Table 4 and Table 5 show the action recognition accuracy on Kinetics and NTU RGB+D, respectively.

From Table 4 we can see that, compared with AAM-GCN, AAM-GCN wo/adap has degradations of 1.59% and 1.83% for top-1 and top-5 accuracies on Kinetics. For the NTU RGB+D dataset, AAM-GCN wo/adap gets much lower accuracies on Cross-Subject of 82.09% and Cross-View of 89.32% compared with AAM-GCN in Table 5. However, it is still better than ST-GCN [20] in the corresponding item. The results indicate that the original structure of the human skeleton graph in ST-GCN has limitations in describing actions. Joints are moving collaboratively in a group when performing actions. That is, although some joints do not have a physical connection, they have strong correlations for some actions, which is ignored in the original graph structure of ST-GCN.

Moreover, the elements in the original adjacency matrix that were set to zero in ST-GCN would never be changed during the training process, which means these joint pairs would never establish relations in any actions. The adaptive graph structure in AAM-GCN can resolve this problem by adding an adaptive matrix $P_{m}$ to rectify the original adjacency, and the elements in $P_{m}$ can be adjusted adaptively during training.

**Figure 8:**
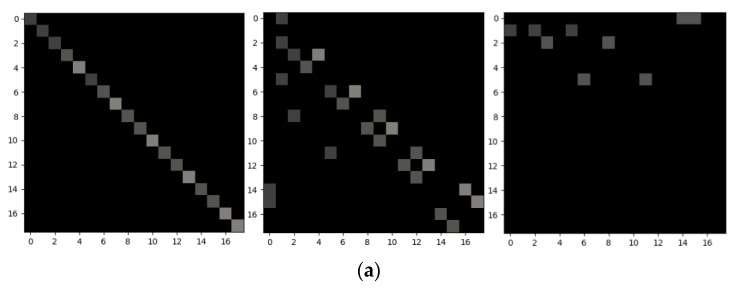

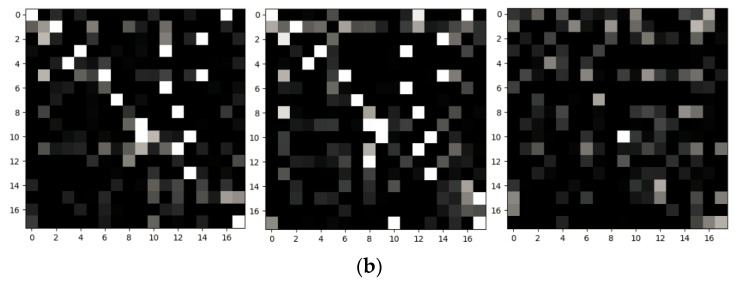
Visualization of the adjacent matrix of each subset in the Kinetics dataset. (**a**) The original adjacent matrix employed in ST-GCN [20]. (**b**) The adaptive adjacency matrix learned by our model.

**Figure 9:**
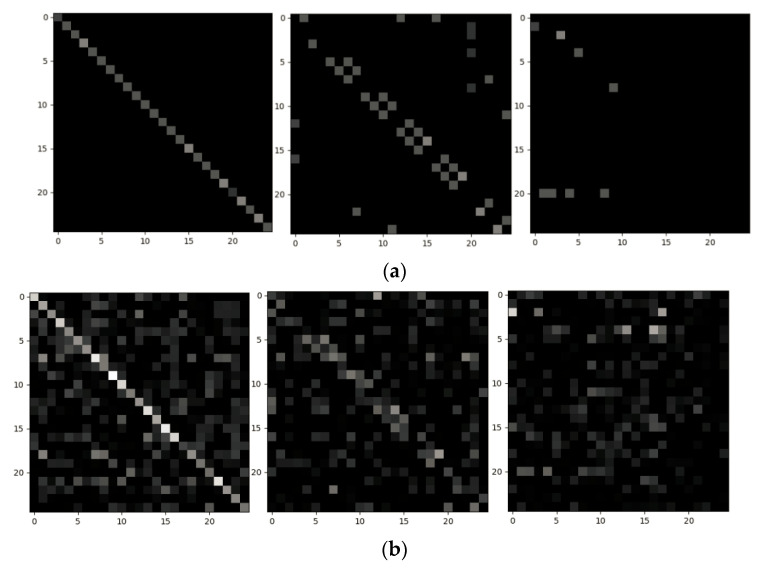
Visualization of the adjacent matrix of each subset in the NTU RGB+D dataset (Cross-View benchmark). (**a**) The original adjacent matrix employed in ST-GCN [20]. (**b**) The adaptive adjacency matrix learned by our model.

To intuitively demonstrate the structure of the adjacency matrix, we visualize the original adjacency matrix employed in ST-GCN and the adaptive adjacency matrix learned in AAM-GCN. Figure 8 and Figure 9 show the different adjacency matrices of each subset on the Kinetics and NTU RGB+D datasets of Cross-View. The spatial configuration partitioning strategy is adopted as in [20], so the neighbor set will be divided into M=3 subsets. From Figure 8a and Figure 9a we can see that the relations are only established between the joints with a physical connection in the original adjacency matrix. Figure 8b and Figure 9b show the corresponding adaptive adjacency matrix. It is obvious to see that the learned structure of the graph in AAM-GCN is more flexible, which can establish relations between any joints performing collaborative actions.

#### 5.4.2. Effect of Attention Mechanism

To validate the impact of the attention mechanism on improving recognition accuracy, we remove the attention part from AAM-GCN termed as AAM-GCN wo/att, which means without attention. Therefore, the memory ${ℳ}^{\left(n\right)}$ will not be multiplied by the attention matrix $F^{\left(n\right)}$, and ${ℳ}_{F}^{\left(n\right)}$ is substituted by ${ℳ}^{\left(n\right)}$ in Equation (9). From Table 4 we can see that AAM-GCN wo/att has a much lower performance of 32.11% compared with AAM-GCN of 33.12% of top-1 on Kinetics. The accuracy of top-5 also has a decrease of 2.01%. The experiment results of AAM-GCN wo/att on NTU RGB+D are shown in Table 5. The accuracies decrease on Cross-Subject and Cross-View with 1.01% and 1.37%, respectively. The attention could help the model to gradually focus on key frames according to the hidden states of GRU because the intermediate hidden states contain lots of temporal dynamics information and spatial configuration information.

#### 5.4.3. Effect of Bidirectional Memory

For the action recognition task, it is important to remember the context information. GRU has the ability to construct temporal relations and capture useful long-range dependencies, which is beneficial for action recognition. In order to evaluate the effectiveness of bidirectional memory, we only use forward GRU instead of bidirectional GRU to construct the memory module. The degraded method is termed as AAM-GCN wo/bidire, which means AAM-GCN without bidirection. Therefore, the item $G_{B}\left(X_{s}\right)$ in Equation (7) is set to 0. The results are shown in Table 4 and Table 5 for Kinetics and NTU RGB+D, respectively. In Table 4, AAM-GCN wo/bidire yields 32.27% of top-1 accuracy on Kinetics. Compared with bidirectional memory, it has a decrease of 0.85%. It also gets lower accuracies in the NTU RGB+D dataset, as shown in Table 5. Compared with AAM-GCN, AAM-GCN wo/bidire decreases 0.88% on Cross-Subject and 1.09% on Cross-View. The results demonstrate that not only what happened in the past is helpful, but also the future information is important for action recognition. Therefore, the bidirectional GRU is effective in building the temporal memory.

#### 5.4.4. Effect of ASGC Concatenation

In order to extract effective temporal information without weakening the spatial configuration information, the spatial feature computed by ASGC is concatenated to the temporal feature computed by attention memory as the final feature for classification, as shown in Figure 3. For evaluating the effectiveness of the spatial feature combination, we remove the concatenation and only use the output of the attention memory module for final recognition. The degraded model is termed as AAM-GCN wo/con, which means AAM-GCN without spatial feature concatenation. It is evaluated in both Kinetics and NTU RGB+D datasets. From Table 4 we can see that the recognition accuracies are 31.81% of top-1 and 54.17% of top-5 on Kinetics. Compared with AAM-GCN, it decreases 1.31% and 1.48%, respectively. For the NTU RGB+D dataset, the degradation is 0.59% and 0.87% for Cross-Subject and Cross-View, respectively, as shown in Table 5. It demonstrates that the concatenation of spatial features is necessary and beneficial for recognition.

#### 5.4.5. Other Parameters Evaluation

We also evaluate the potential parameters that are set in the architecture in the NTU RGB+D dataset. The attention memory module is constructed by multi-bidirectional GRU layers. The number of layers is evaluated, and the results are shown in Table 6. It is observed that increasing the number of layers can improve the performance (adopting two and three layers yields better results compared with only one layer). Nevertheless, the more layers there are does not make the performance better (the accuracy is decreased when adopting three layers compared with two layers). The degradation is caused by over-fitting since introducing new layers would bring more parameters. Consequently, we do not try more layers due to the GPU’s memory limitation.

According to the above experiments, the attention memory module is finally constructed with two bidirectional GRU layers. The number of hidden neurons in each layer is evaluated via Kinetics, and the results are shown in Table 7. We can see that it gets much better results when the numbers of hidden neurons of the two layers are set to 128 and 64.

## 6. Conclusions and Future Work

In this paper, we proposed an Adaptive Attention Memory Convolutional Network (AAM-GCN) for skeleton action recognition. We designed an adaptive adjacent matrix to construct the spatial graph and adopt graph convolution to extract spatial features. The adaptive adjacent matrix will be optimized in a data-driven manner according to the actions during the training process and will better describe the actions in the real world. To extract the inter-frame action features, we constructed an attention-enhanced memory via bidirectional GRU. It can effectively capture temporal relations by the attention-enhanced memory. Due to the bidirectional characteristics, it can not only remember what happened in the past but also employ the information for the future. We evaluate our model on three large datasets: NTU RGB+D, Kinetics and HDM05. The experimental results demonstrate that our AAM-GCN achieves better performance than some other state-of-the-art methods.

However, there are still some limitations of our method. For the action recognition task, the scene and the objects that interacted with the person play an important role. This useful information is ignored by the skeleton data, which only employed the location information of joints. There are also redundant joints in the skeleton that could be merged to reduce the complexity and make the graph structure more robust. The future work includes the following issues: constructing a more general network architecture for different types of action recognition. Other auxiliary information, such as the objects that the person interacted with, and the scene where the action took place, would contribute to the action recognition task. Further study in action anticipating is also considered in future work.

## Figures and Tables

**Table 2:** Comparison results of various methods on the NTU RGB+D dataset.

Methods	Cross Subject (%)	Cross View (%)
ST-LSTM [6]	69.20	77.70
Two-stream RNN [15]	71.30	79.50
GCA-LSTM [12]	74.40	82.80
Visualization CNN [35]	76.00	82.60
VA-LSTM [39]	79.40	87.60
Clips+CNN+MTLN [36]	79.57	84.83
ST-GCN [20]	81.50	88.30
3D-GCN [56]	82.60	89.60
DS-LSTM [57]	77.80	87.33
AAM-GCN (ours)	82.73	90.12

**Table 3:** Comparison results of various methods on the HDM05 dataset.

Methods	Acc (%)
Multi-layer Perceptron [53]	95.59
Hierarchical RNN [11]	96.92
ST-GCN [20]	96.50
Deep LSTM+Co-occurrence+In-depth Dropout [40]	97.25
AS-GCN [17]	97.40
AAM-GCN (ours)	98.30

**Table 4:** Ablation experiments on the Kinetics dataset. “ST-GCN” [20] is compared as a baseline method. For the setting of each method, please refer to Section 5.4.

Methods	Top-1 Acc (%)	Top-5 Acc (%)
ST-GCN [20]	30.70	53.80
AAM-GCN wo/adap	31.53	53.82
AAM-GCN wo/att	32.11	53.64
AAM-GCN wo/bidire	32.27	54.50
AAM-GCN wo/con	31.81	54.17
AAM-GCN (ours)	33.12	55.65

**Table 5:** Ablation experiments on the NTU RGB+D dataset. “ST-GCN” [20] is compared as a baseline method. For the setting of each method, please refer to Section 5.4.

Methods	Cross Subject (%)	Cross View (%)
ST-GCN [20]	81.50	88.30
AAM-GCN wo/adap	82.09	89.32
AAM-GCN wo/att	81.72	88.75
AAM-GCN wo/bidire	81.85	89.03
AAM-GCN wo/con	82.34	89.65
AAM-GCN (ours)	82.73	90.12

**Table 6:** Comparison of different bidirectional GRU layers in the NTU RGB+D dataset.

Number of Bidirectional GRU Layers	Cross Subject (%)	Cross View (%)
1	81.85	89.43
2	82.73	90.12
3	82.52	90.03

**Table 7:** Different numbers of hidden neurons in each bidirectional layer. The evaluation is taken in the Kinetics dataset.

Number of Hidden Neurons		Top-1 Acc (%)	Top-5 Acc (%)
1st Layer	2nd Layer		
256	128	32.70	54.30
128	64	33.12	55.65
64	32	31.67	54.75

## Data Availability

NTU RGB+D: https://rose1.ntu.edu.sg/dataset/actionRecognition/. Skeleton data of Kinetics: https://github.com/yysijie/st-gcn/blob/master/OLD_README.md. HDM05: http://resources.mpi-inf.mpg.de/HDM05/.

## References

[1] Hu J., Zhu E., Wang S., Liu X., Guo X., Yin J. (2019). An Efficient and Robust Unsupervised Anomaly Detection Method Using Ensemble Random Projection in Surveillance Videos. Sensors.

[2] Duric Z., Gray W.D., Heishman R., Li F., Rosenfeld A., Schoelles M.J., Schunn C., Wechsler H. (2002). Integrating perceptual and cognitive modeling for adaptive and intelligent human-computer interaction. Proc. IEEE.

[3] Sudha M.R., Sriraghav K., Abisheck S.S., Jacob S.G., Manisha S. (2017). Approaches and applications of virtual reality and gesture recognition: A review. Int. J. Ambient. Comput. Intell..

[4] Simonyan K., Zisserman A. Two-stream Convolutional Networks for Action Recognition in Videos. Proceedings of the 27th International Conference on Neural Information Processing Systems.

[5] Wang L., Xiong Y., Wang Z., Qiao Y., Lin D., Tang X., Van Gool L. Temporal Segment Networks: Towards Good Practices for Deep Action Recognition. Proceedings of the European Conference on Computer Vision.

[6] Liu J., Shahroudy A., Xu D., Wang G. Spatio-temporal LSTM with trust gates for 3D human action recognition. Proceedings of the 14th European Conference on Computer Vision (ECCV).

[7] Han F., Reily B., Hoff W., Zhang H. (2017). Space-time Representation of People based on 3D Skeletal Data: A Review. Comput. Vis. Image Underst..

[8] Presti L.L., La Cascia M. (2016). 3D skeleton-based human action classification: A survey. Pattern Recognit..

[9] Cao Z., Simon T., Wei S.E., Sheikh Y. (2017). Realtime Multi-Person 2D Pose Estimation using Part Affinity Fields. arXiv.

[10] Fang H.S., Xie S., Tai Y.W., Lu C. (2017). RMPE: Regional Multi-Person Pose Estimation. ICCV. https://github.com/MVIG-SJTU/AlphaPose.

[11] Du Y., Wang W., Wang L. Hierarchical recurrent neural network for skeleton based action recognition. Proceedings of the IEEE Conference on Computer Vision and Pattern Recognition.

[12] Liu J., Wang G., Hu P., Duan L.Y., Kot A.C. Global context-aware attention lstm networks for 3d action recognition. Proceedings of the 2017 IEEE Conference on Computer Vision and Pattern Recognition (CVPR).

[13] Xie C., Li C., Zhang B., Chen C., Han J., Zou C., Liu J. (2018). Memory attention networks for skeleton-based action recognition. arXiv.

[14] Fernando B., Gavves E., Oramas J.M., Ghodrati A., Tuytelaars T. Modeling Video Evolution for Action Recognition. Proceedings of the 2015 IEEE Conference on Computer Vision and Pattern Recognition (CVPR).

[15] Wang H., Wang L. Modeling temporal dynamics and spatial configurations of actions using two-stream recurrent neural networks. Proceedings of the IEEE Conference on Computer Vision and Pattern Recognition.

[16] Vemulapalli R., Arrate F., Chellappa R. Human action recognition by representing 3d skeletons as points in a lie group. Proceedings of the IEEE Conference on Computer Vision and Pattern Recognition.

[17] Li M., Chen S., Chen X., Zhang Y., Wang Y., Tian Q. Actional-structural graph convolutional networks for skeleton-based action recognition. Proceedings of the IEEE Conference on Computer Vision and Pattern Recognition.

[18] Si C., Chen W., Wang W., Wang L., Tan T. An attention enhanced graph convolutional lstm network for skeleton-based action recognition. Proceedings of the IEEE Conference on Computer Vision and Pattern Recognition.

[19] Shi L., Zhang Y., Cheng J., Lu H. Two-stream adaptive graph convolutional networks for skeleton-based action recognition. Proceedings of the IEEE Conference on Computer Vision and Pattern Recognition.

[20] Yan S., Xiong Y., Lin D. Spatial temporal graph convolutional networks for skeleton-based action recognition. Proceedings of the AAAI Conference on Artificial Intelligence.

[21] Kipf T.N., Welling M. (2016). Semi-Supervised Classification with Graph Convolutional Networks. arXiv.

[22] Niepert M., Ahmed M., Kutzkov K. Learning Convolutional Neural Networks for Graphs. Proceedings of the International Conference on Machine Learning.

[23] Monti F., Boscaini D., Masci J., Rodola E., Svoboda J., Bronstein M.M. Geometric Deep Learning on Graphs and Manifolds Using Mixture Model CNNs. Proceedings of the IEEE Conference on Computer Vision and Pattern Recognition (CVPR).

[24] Li B., Li X., Zhang Z., Wu F. Spatio-temporal graph routing for skeleton-based action recognition. Proceedings of the AAAI Conference on Artificial Intelligence.

[25] Shi L., Zhang Y., Cheng J., Lu H. Skeleton-based action recognition with directed graph neural networks. Proceedings of the IEEE/CVF Conference on Computer Vision and Pattern Recognition.

[26] Wen Y., Gao L., Fu H., Zhang F., Xia S. Graph CNNs with motif and variable temporal block for skeleton-based action recognition. Proceedings of the 33rd AAAI Conference on Artificial Intelligence.

[27] Yang W., Zhang J., Cai J., Xu Z. (2021). Shallow Graph Convolutional Network for Skeleton-Based Action Recognition. Sensors.

[28] Shahroudy A., Liu J., Ng T.T., Wang G. NTU RGB+D: A Large Scale Dataset for 3D Human Activity Analysis. Proceedings of the IEEE Conference on Computer Vision and Pattern Recognition.

[29] Kay W., Carreira J., Simonyan K., Zhang B., Hillier C., Vijayanarasimhan S., Viola F., Green T., Back T., Natsev P. (2017). The Kinetics Human Action Video Dataset. arXiv.

[30] Müller M., Röder T., Clausen M., Eberhardt B., Krüger B.A. (2007). Weber: Documentation Mocap Database HDM05.

[31] Yang Z., Li Y., Yang J., Luo J. (2018). Action Recognition with Spatio-Temporal Visual Attention on Skeleton Image Sequences. IEEE Trans. Circuits Syst. Video Technol..

[32] Baradel F., Wolf C., Mille J. Human Action Recognition: Pose-based Attention Draws Focus to Hands. Proceedings of the 2017 IEEE International Conference on Computer Vision Workshops (ICCVW).

[33] Li C., Zhong Q., Xie D., Pu S. Co-Occurrence Feature Learning from Skeleton Data for Action Recognition and Detection with Hierarchical Aggregation. Proceedings of the Twenty-Seventh International Joint Conference on Artificial Intelligence IJCAI-18.

[34] Kim T.S., Reiter A. Interpretable 3D Human Action Analysis with Temporal Convolutional Networks. Proceedings of the 2017 IEEE Conference on Computer Vision and Pattern Recognition Workshops (CVPRW).

[35] Liu M., Liu H., Chen C. (2017). Enhanced skeleton visualization for view invariant human action recognition. Pattern Recognit..

[36] Ke Q., Bennamoun M., An S., Sohel F., Boussaid F. (2017). A New Representation of Skeleton Sequences for 3D Action Recognition. Proceedings of the 2017 IEEE Conference on Computer Vision and Pattern Recognition (CVPR).

[37] Li W., Wen L., Chang M.C., Lim S.N., Lyu S. Adaptive RNN tree for large-scale human action recognition. Proceedings of the IEEE International Conference on Computer Vision (ICCV).

[38] Song S., Lan C., Xing J., Zeng W., Liu J. (2016). An End-to-End Spatio-Temporal Attention Model for Human Action Recognition from Skeleton Data. arXiv.

[39] Zhang P., Lan C., Xing J., Zeng W., Xue J., Zheng N. View Adaptive Recurrent Neural Networks for High Performance Human Action Recognition From Skeleton Data. Proceedings of the IEEE International Conference on Computer Vision (ICCV).

[40] Zhu W., Lan C., Xing J., Zeng W., Li Y., Shen L., Xie X. Co-occurrence feature learning for skeleton based action recognition using regularized deep LSTM networks. Proceedings of the AAAI Conference on Artificial Intelligence.

[41] Cho K., Merrienboer V.B., Gulcehre C., Bahdanau D., Bougares F., Schwenk H., Bengio Y. (2014). Learning Phrase Representations Using RNN Encoder-Decoder for Statistical Machine Translation. arXiv.

[42] Liu Z., Zhang H., Chen Z., Wang Z. Disentangling and Unifying Graph Convolutions for Skeleton-Based Action Recognition. Proceedings of the 2020 IEEE Conference on Computer Vision and Pattern Recognition (CVPR).

[43] Ye F., Pu S., Zhong Q., Li C. Dynamic GCN: Context-enriched Topology Learning for Skeleton-based Action Recognition. Proceedings of the 28th ACM International Conference on Multimedia.

[44] Plizzari C., Cannici M., Matteucci M. (2021). Spatial Temporal Transformer Network for Skeleton-Based Action Recognition. International Conference on Pattern Recognition.

[45] Chen Y., Zhang Z., Yuan C. Channel-wise Topology Refinement Graph Convolution for Skeleton-Based Action Recognition. Proceedings of the IEEE International Conference on Computer Vision (ICCV).

[46] Sharma S., Kiros R., Salakhutdinov R. (2015). Action recognition using visual attention. arXiv.

[47] Luong M.T., Pham H., Manning C.D. Effective approaches to attention-based neural machine translation. Proceedings of the 2015 Conference on Empirical Methods in Natural Language Processing, EMNLP 2015.

[48] Xu K., Ba J., Kiros R., Cho K., Courville A., Salakhudinov R., Zemel R., Bengio Y. Show, attend and tell: Neural image caption generation with visual attention. Proceedings of the 32nd International Conference on Machine Learning.

[49] Yao L., Torabi A., Cho K., Ballas N., Pal C., Larochelle H., Courville A. Describing videos by exploiting temporal structure. Proceedings of the 2015 IEEE International Conference on Computer Vision (ICCV).

[50] Stollenga M.F., Masci J., Gomez F., Schmidhuber J. Deep networks with internal selective attention through feedback connections. Proceedings of the Annual Conference on Neural Information Processing Systems 2014.

[51] Wang Y., Wang S., Tang J., O’Hare N., Chang Y., Li B. (2016). Hierarchical Attention Network for Action Recognition in Videos. arXiv.

[52] Goodfellow I., Bengio Y., Courville A. (2016). Deep Learning. MIT Press. http://www.deeplearningbook.org.

[53] Cho K., Chen X. Classifying and Visualizing Motion Capture Sequences using Deep Neural Networks. Proceedings of the 2014 International Conference on Computer Vision Theory and Applications (VISAPP).

[54] Peng W., Shi J., Varanka T., Zhao G. (2021). Rethinking the ST-GCNs for 3D skeleton-based human action recognition. Neurocomputing.

[55] Pishchulin L., Insafutdinov E., Tang S. Deepcut: Joint Subset Partition and Labeling for Multi Person Pose Estimation. Proceedings of the 2016 IEEE Conference on Computer Vision and Pattern Recognition (CVPR).

[56] Gao X., Li K., Miao Q., Sheng L. 3D Skeleton-Based Video Action Recognition by Graph Convolution Network. Proceedings of the 2019 IEEE International Conference on Smart Internet of Things (SmartIoT).

[57] Jiang X., Xu K., Sun T. (2020). Action Recognition Scheme Based on Skeleton Representation with DS-LSTM Network. IEEE Trans. Circuits Syst. Video Technol..

